# Chest wall abscess and costochondritis due to *Salmonella enterica* serotype Choleraesuis: a case report

**DOI:** 10.1186/s13256-023-04229-w

**Published:** 2023-12-06

**Authors:** Nassim R. Mokraoui, Rose Ganim, Armando Paez

**Affiliations:** 1 Division of Infectious Diseases, UMass Chan Medical School-Baystate, Springfield, MA 01199 USA; 2 Division of Thoracic Surgery, UMass Chan Medical School-Baystate, Springfield, MA 01199, USA

**Keywords:** *Salmonella enterica* serotype Choleraesuis, Costochondritis, Osteomyelitis, Extra-abdominal infection

## Abstract

**Background:**

*Salmonella enterica* serotype Choleraesuis infections usually cause self-limited gastrointestinal diseases. Extra-abdominal infections are often secondary to bacteremia in immunocompromised individuals and are relatively rare in immunocompetent hosts.

**Case presentation:**

A 65-year-old Caucasian female initially presented to the thoracic surgery clinic due to a poorly healing wound on her chest. Her condition started after a mechanical fall hitting her chest with interval development of a tender lump that later spontaneously drained. A chest computed tomography scan with intravenous contrast demonstrated an abnormal infiltration with small foci of fluid and air consistent with a small abscess anterior to the left seventh costal cartilage. Aspirate culture of the abscess grew *S. enterica* serotype Choleraesuis susceptible to ampicillin and trimethoprim/sulfamethoxazole. The patient had no prior history of signs or symptoms of gastrointestinal infection. Blood cultures were negative. With a background of penicillin allergy, she was treated with trimethoprim/sulfamethoxazole, and later with ceftriaxone due to persistent drainage of the wound. Follow-up chest computed tomography scan with intravenous (IV) contrast showed continued abnormal findings previously seen in the computed tomography scan with the appearance of a sinus tract. The patient subsequently underwent surgical debridement and partial resection of the left seventh costochondral cartilage and excision of the fistula. She had an uneventful recovery and complete resolution of her condition.

**Conclusion:**

We report a rare case of chest wall abscess with associated costochondritis due to *S. enterica* serotype Choleraesuis in a patient with no evidence of immunodeficiency nor history of bacteremia. Extraintestinal infections due to *Salmonella* without documented bacteremia have been previously reported in the literature. History of local trauma to the affected area might contribute to the seeding of infection. Diagnosis is often accomplished by clinical evaluation and culture of the affected area. Treatment often involves targeted antibiotic therapy but may require surgical intervention to achieve source control and cure.

## Introduction

*S. enterica * serotype Choleraesuis, a nontyphoidal *Salmonella*, often causes self-limited gastrointestinal disease with rare extra-abdominal involvement. However, this organism can be invasive, with documented bacteremia and little intestinal involvement [1]. The CDC has also reported a significantly higher number of this serotype isolated from the blood than stool in all isolates evaluated [2]. Bacteremia was shown to have a higher incidence in immunosuppressed patients [3]. Dissemination to the bones and cartilage causing osteomyelitis and costochondritis has been reported since 1937 [4, 5]. Here, we report a case of *S. enterica* serotype Choleraesuis chest wall abscess and costochondritis in a patient with a history of local trauma to the chest without documented bacteremia or associated risk factors for disseminated infection.

## Case

**Fig. 1:**
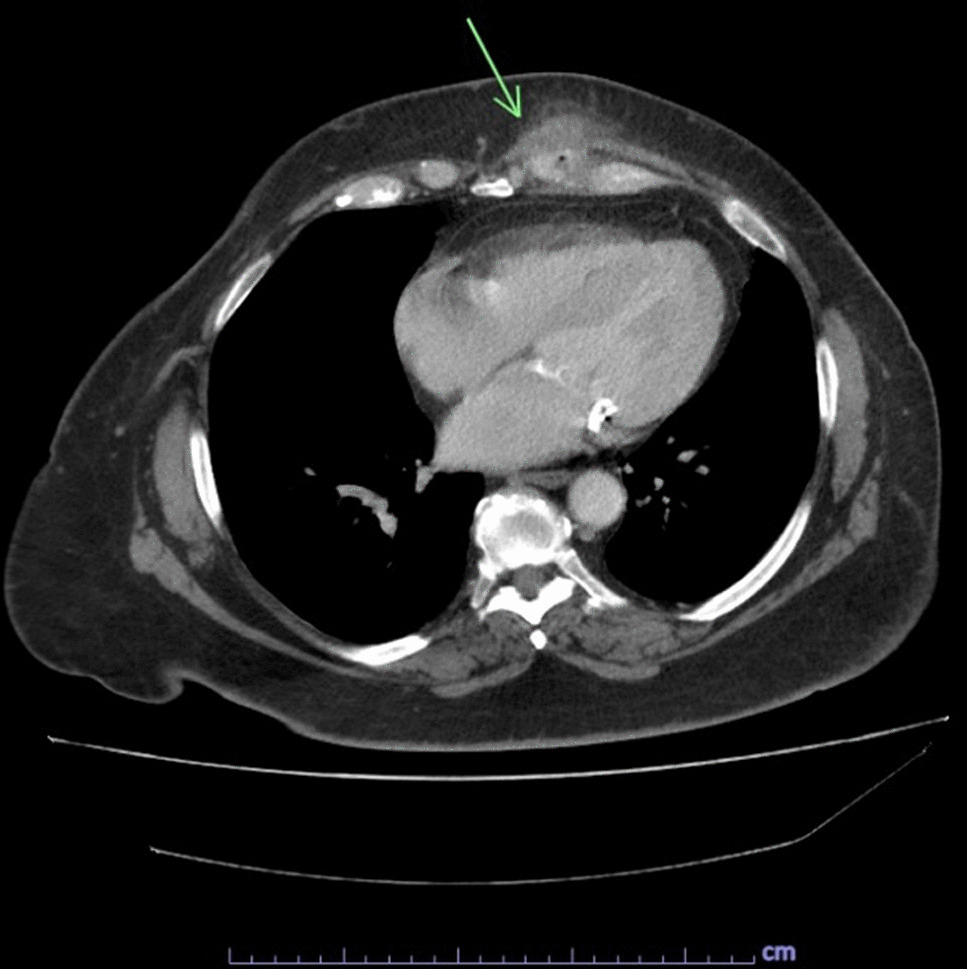
Chest Computed Tomography with Intravenous contrast, 11 March 2022. Arrow demonstrates phlegmon with fluid collection involving seventh costal cartilage

**Fig. 2:**
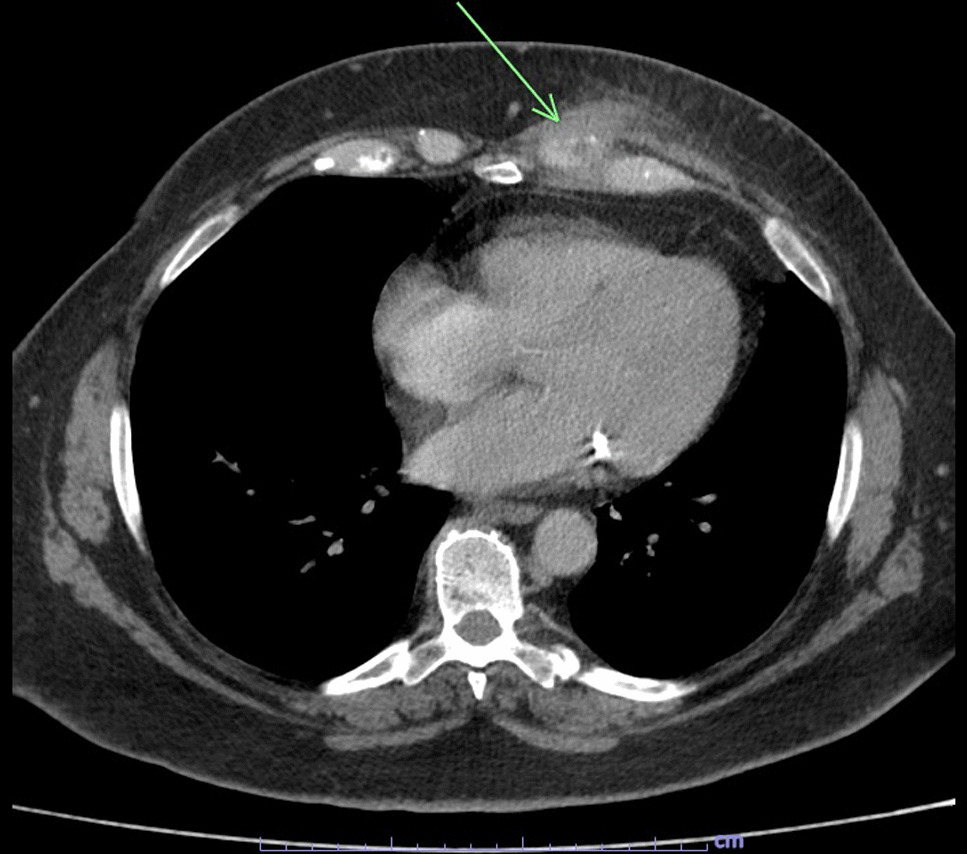
Chest Computed Tomography with Intravenous contrast, 11 April 2022. Arrow demonstrates persistent phlegmon and fluid collection involving seventh costal cartilage

**Fig. 3:**
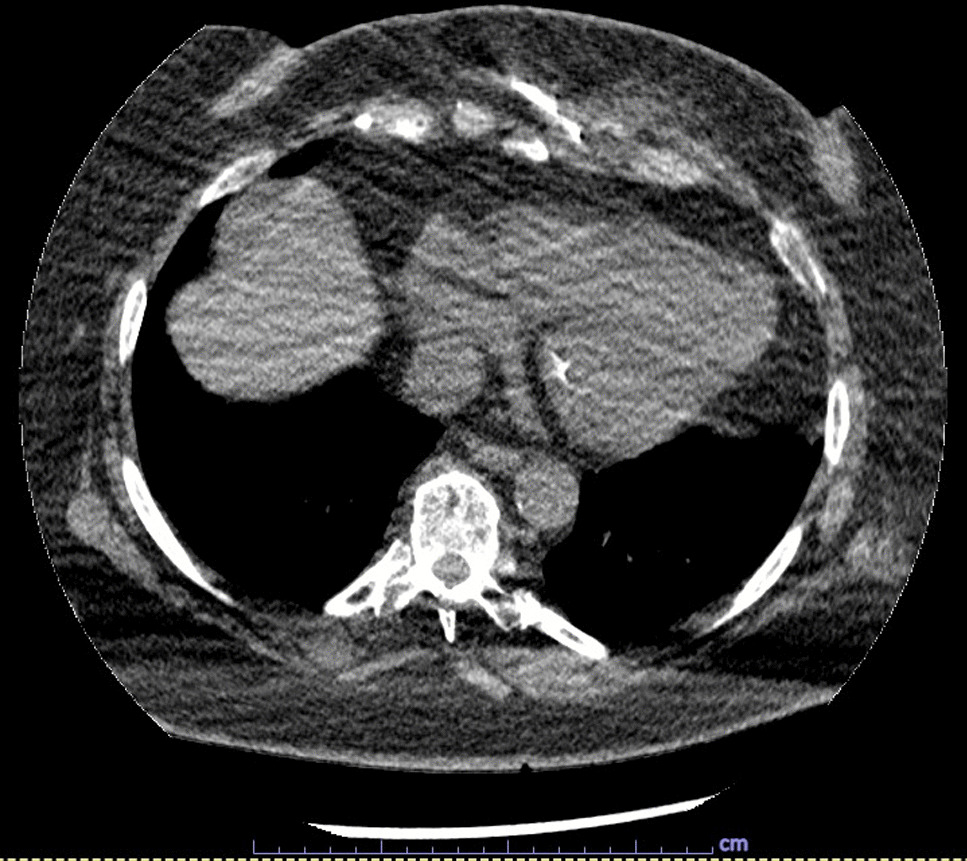
Computed Tomography-guided needle aspirate, 25 April 2022

The patient is a 65-year-old Caucasian female G0P0 with a past medical history of meningioma status post resection of a left parieto-occipital meningioma in 2011 with residual impairment in her left peripheral vision and hypertension, well-controlled diabetes mellitus, gastroesophageal reflux, and difficulty with ambulation who developed a chronic draining wound in the left chest wall area. About 4 months before clinical presentation, she was diagnosed with coronavirus disease 2019 (COVID-19) pneumonia, for which she received sotrovimab, a COVID-19 monoclonal antibody (500 mg/IV as a single dose), a 5-day course of azithromycin (500 mg per mouth on day 1, and then 250 mg per mouth daily for days 2–5), and prednisone 20 mg per mouth daily for 5 days. Three months before her presentation, she fell, hitting her left anterior chest against the wooden footboard of her bed without incurring any wound. Shortly thereafter, she noticed a sore lump in that region that progressively increased in size. A month later, she presented to her primary care provider with subjective fevers, chills, and night sweats. She was prescribed with doxycycline orally at 100 mg every 12 hours for 7 days with no resolution of her symptoms. Laboratory tests showed a white blood cell (WBC) count of 9.2 k/mm3, Hgb A1c of 5.6%, and normal liver and kidney function tests. A chest computed tomography (CT) scan with intravenous contrast demonstrated an abnormal infiltrate with small foci of fluid and air measuring 6–7 mm, concerning for a small abscess along the anterior left seventh costal cartilage (Fig. 1). Subsequently, she was referred to thoracic surgery, where a chest ultrasound showed a 1 cm fluid collection, 3 cm deep in the soft tissue. A 1 ml cloudy fluid was drained by ultrasound-guided aspiration and was sent for Gram stain and culture, which were negative. On clinical follow-up 2 weeks later, the patient was treated empirically with clindamycin 300 mg orally three times a day for 21 days to cover for possible anaerobic infection. After antibiotic course completion, a repeat chest CT scan with intravenous contrast showed unchanged findings with mild worsening of the overlying skin thickening. A subcentimeter focus of hypoattenuation within the chondral portion of the left seventh rib was again demonstrated (Fig. 2). The patient underwent repeat CT-guided aspiration for histopathology and cultures—bacterial, fungal, and acid-fact bacteria (AFB) (Fig. 3). All cultures came back with negative Gram stain and growth, and histopathology findings showed fibroconnective tissue with chronic inflammatory changes and dense fibrosis. On subsequent follow-up with thoracic surgery, the biopsy site was noted with draining purulent fluid sent for culture. She was empirically prescribed with cephalexin 500 mg four times a day for 2 weeks for the suspected costochondritis with abscess. The Gram stain of the drainage fluid showed Gram-negative rods and the culture grew *S. enterica* serotype Choleraesuis. The patient was referred to the infectious disease clinic for further evaluation and management recommendations.

**Fig. 4:**
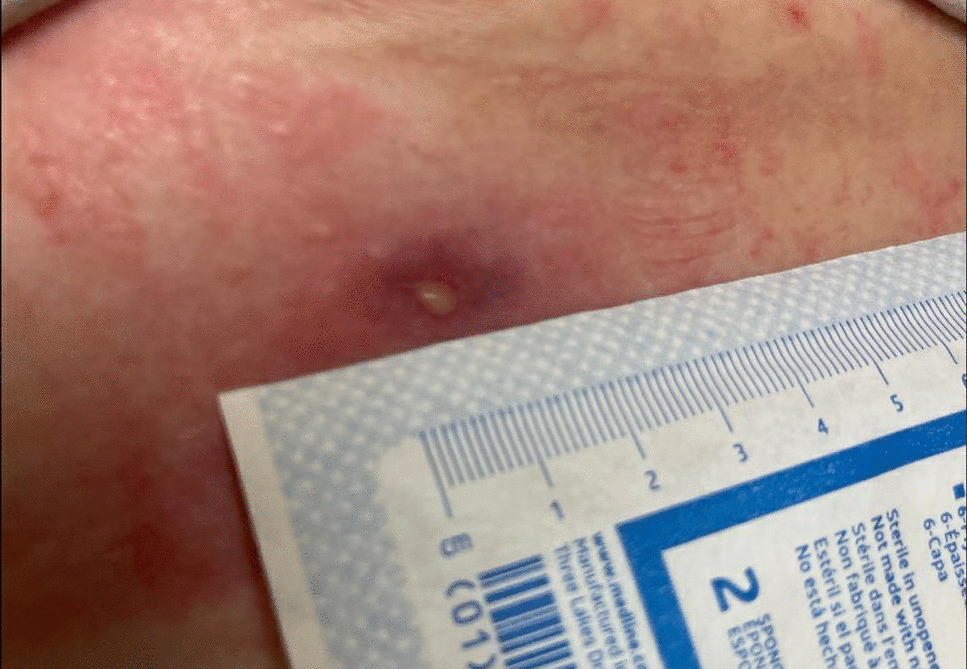
Chest photo showing initial evaluation in our clinic, May 2022

During the infectious disease evaluation, a more detailed history was obtained. The patient did not have any risk factors for disseminated *Salmonella* infection such as chronic steroid use, autoimmune diseases, or malignancies. However, the patient did receive a short course of prednisone when she had COVID-19. The patient denied having any history of gastrointestinal symptoms including nausea, vomiting, diarrhea, or abdominal pain. There were no known animal exposures, including reptiles. No recent history of travel or known sick contacts. The patient denied changes in eating habits or eating raw or reportedly contaminated meats or vegetables. The patient is married and works as an emergency department nurse. She has no history of alcohol, tobacco, or illicit drug use. On examination, she was afebrile with a normal pulse and blood pressure and oxygen saturation on room air. She was in no acute distress and had obesity. She had diminished vision in her left periphery. Pupils were equal, round, reactive to light, and appropriately accommodating. Oral exam revealed good dentition with no posterior erythema or thrush. Her neck exam showed no jugular venous distention, cervical adenopathy, or thyromegaly. The left anterior chest area showed an open wound with a pinhole-size opening located inferior to her breast with associated local swelling and serous wound discharge (Fig. 4). Auscultation of the lungs was clear and with good chest expansion. Cardiac auscultation revealed a regular rate and rhythm with no apparent murmurs, rubs, or gallops. Lower extremity pulses were normal. The abdomen was soft, nontender, nondistended, and with normal bowel sounds. Musculoskeletal and joint examinations were unremarkable. Skin examination was without rash, ulcers, excoriations, or jaundice. A neurological exam revealed her known ambulation issues, but no reflex or sensation abnormalities were noted. A repeat complete blood count (CBC) showed a WBC count of 7.2 k/mm3 and elevated c-reactive protein (CRP) of 1.0 mg/dL (reference range: 0–0.5 mg/dL). Patient hemoglobin, platelets, and comprehensive metabolic panel (CMP) were all within normal limits. A repeat culture of the wound discharge was again obtained. A Gram stain showed Gram-negative rods and the culture grew *S. enterica* serotype Choleraesuis. Aerobic and anaerobic blood cultures were obtained from separate sites which were negative.

**Fig. 5:**
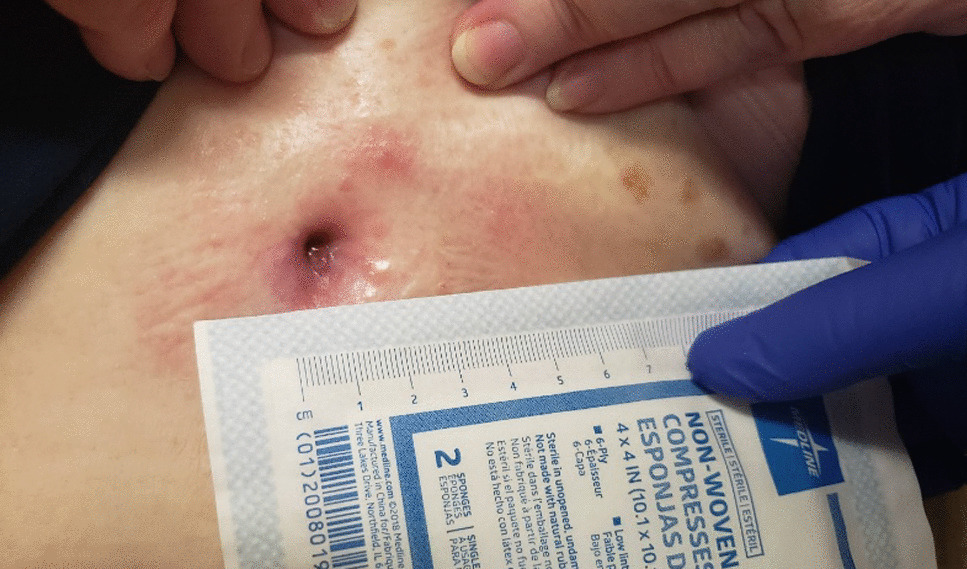
Chest photo demonstrating marginal-to-no improvement in wound, September 2022

**Fig. 6:**
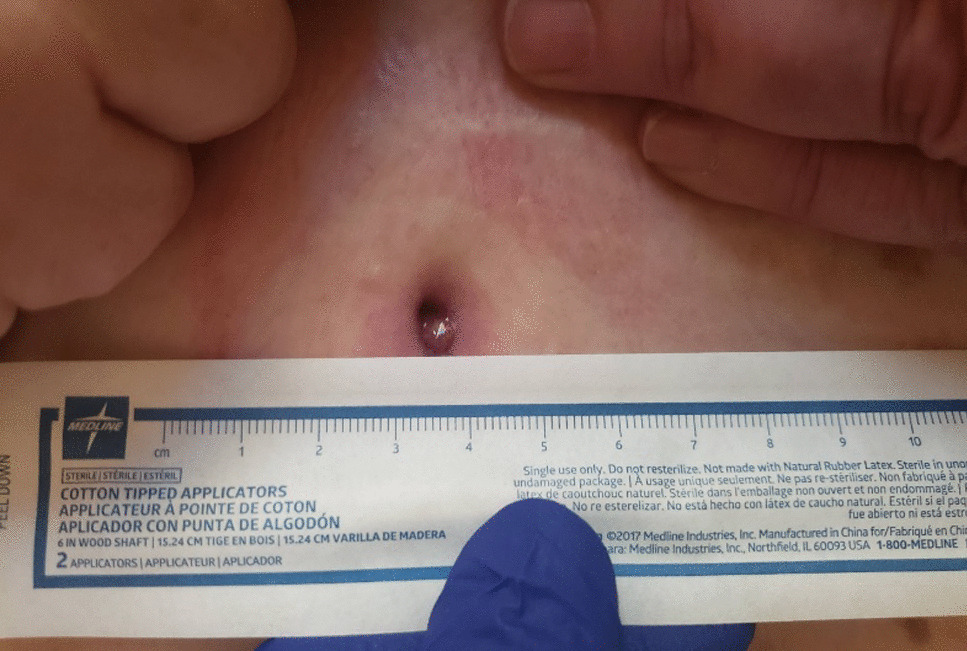
Chest photo of unimproved wound on follow-up in our clinic, October 2022

**Fig. 7:**
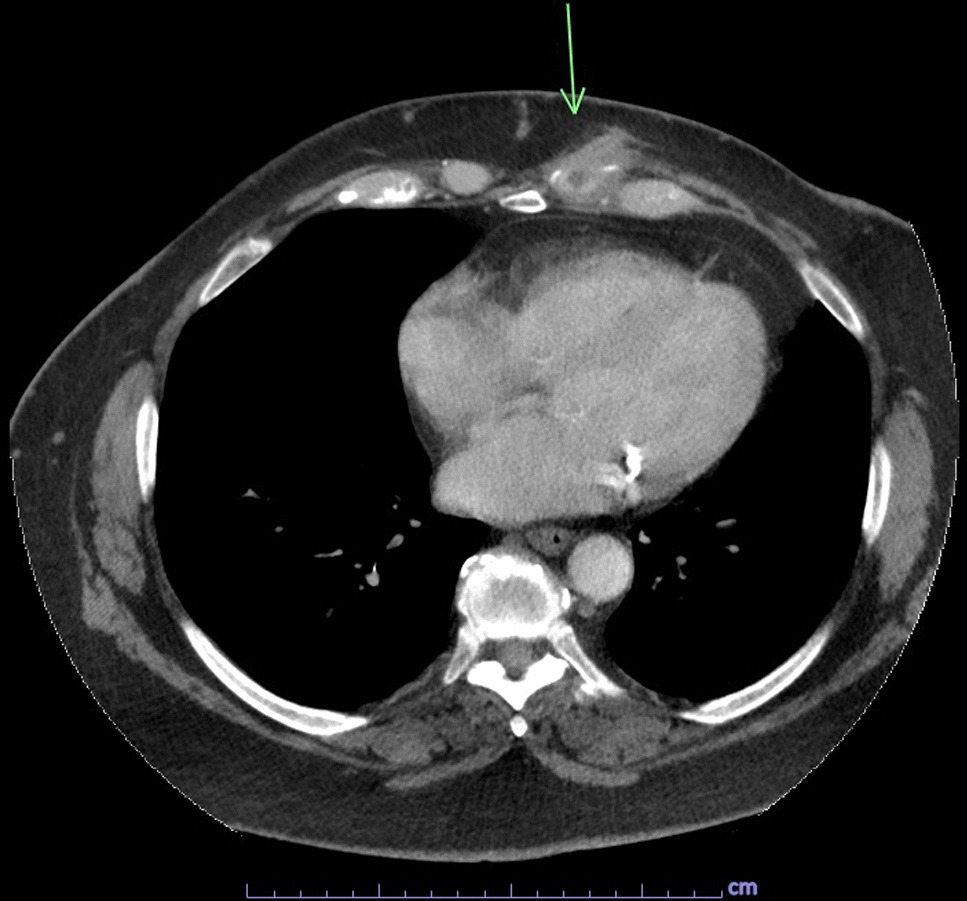
Chest Computed Tomography with Intravenous contrast, 6 October 2022. Arrow demonstrates persistent fluid collection involving seventh costal cartilage

Due to her penicillin allergy, large body habitus, and concern for deep infection with costochondritis and abscess, she was started on trimethoprim–sulfamethoxazole at two double-strength tablets (160 mg TMP and 800 SMX) taken orally twice a day. The dosing was based on her adjusted body weight of 85 kg (4 mg/kg/dose every 12 hours, equating to 340 mg/dose). The antibiotic susceptibility test confirmed susceptibility to both ampicillin and trimethoprim–sulfamethoxazole. The plan was to treat for at least 8 weeks of antibiotic therapy. The follow-up evaluation showed a persistent wound with decreased drainage 3 months later (Figs. 5 and 6). Her clinical course was complicated by the development of a diffuse, pruritic skin rash presumably from trimethoprim–sulfamethoxazole, which resolved upon cessation of the trimethoprim–sulfamethoxazole therapy. Treatment was changed to cefpodoxime (400 mg every 12 hours) until a peripherally inserted central catheter (PICC) line could be placed for ceftriaxone (1 g intravenously daily). Repeat wound cultures were obtained that grew methicillin-resistant *Staphylococcus aureus* susceptible to doxycycline with which she was treated at a dose of 100 mg orally every 12 hours. Repeat chest CT scan with IV contrast showed persistent focal soft tissue thickening surrounding the left seventh rib cartilage extending to the skin with a sinus tract. There was also a redemonstration of a focus of hypoattenuation measuring approximately 0.9 × 0.6 cm near the seventh rib cartilage, consistent with an abscess described in the prior study (Fig. 7). Thoracic surgery recommended surgical intervention for source control of the infection. Subsequently, the patient underwent surgical debridement with excision of the fistula and a partial resection of the left seventh costochondral cartilage. Operative bone tissue was obtained and Gram stain and cultures were negative. Histopathology showed findings consistent with costochondritis. The surgical wound eventually healed postoperatively, and antibiotic therapy was later discontinued after achieving adequate source control of infection. Continued follow-up with cardiothoracics and infectious disease at 6 months and 1 year showed a well-healed wound and without active signs of infection. Her follow-up labs showed a normal CBC, CMP, erythrocyte sedimentation rate (ESR), and CRP at 6- and 12-month follow-up.

During the course of her illness, the patient expressed disappointment and concerns about the nonhealing nature of the chest wound and poor response to prolonged antibiotic treatment. She also expressed concern about the drug rash from trimethoprim–sulfamethoxazole use, but felt relieved when the rash resolved. The patient was cooperative during the treatment and was eventually satisfied with the full resolution of her condition after surgery.

## Discussion

**Fig. 8:**
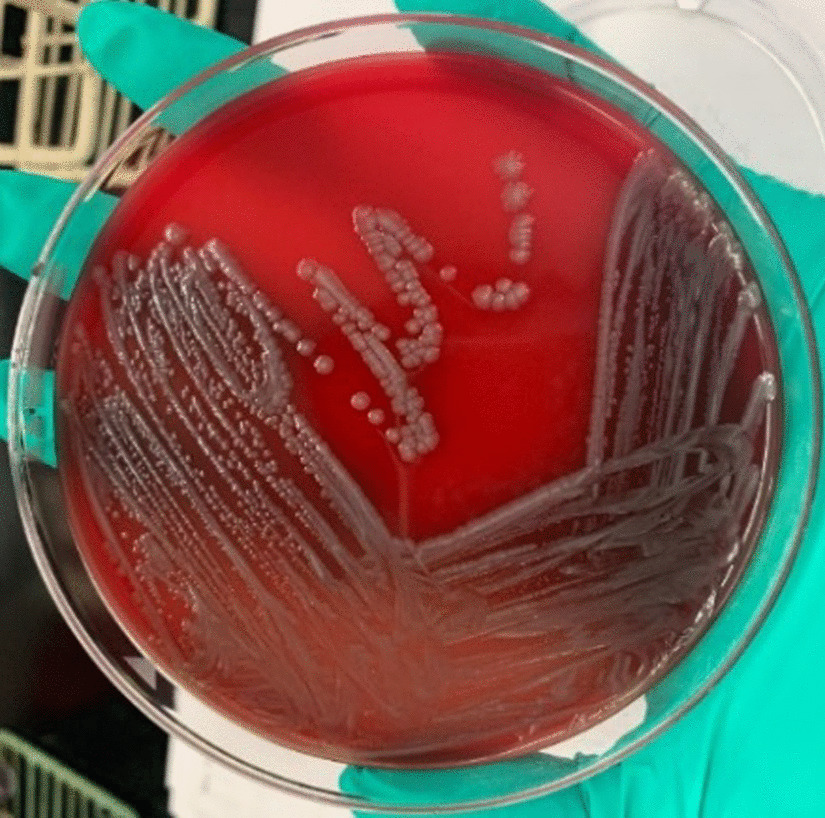
Typical *Salmonella* spp. colonies (blood agar)

**Fig. 9:**
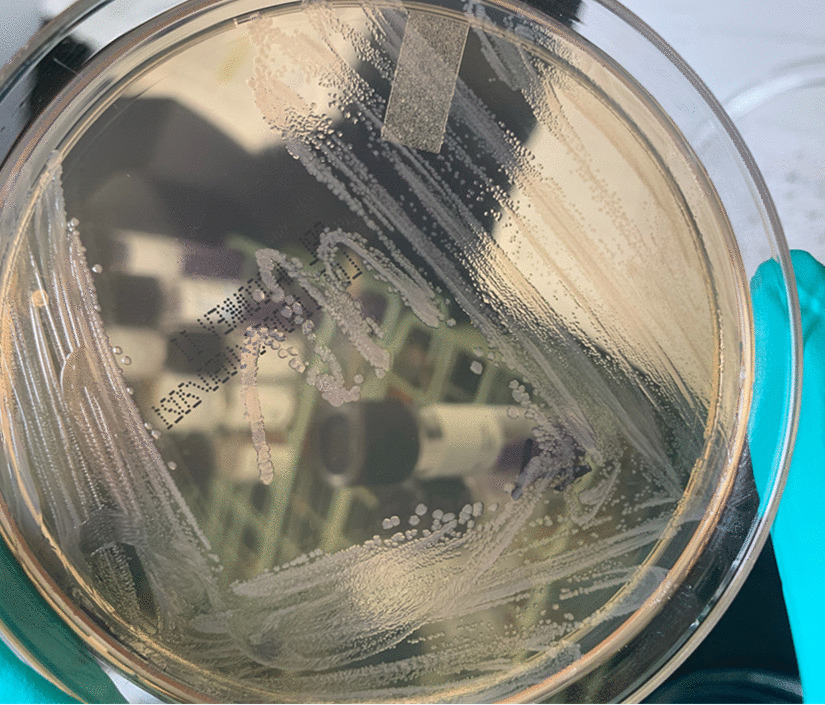
*Salmonella* spp. in selective medium (nutrient agar)

**Fig. 10:**
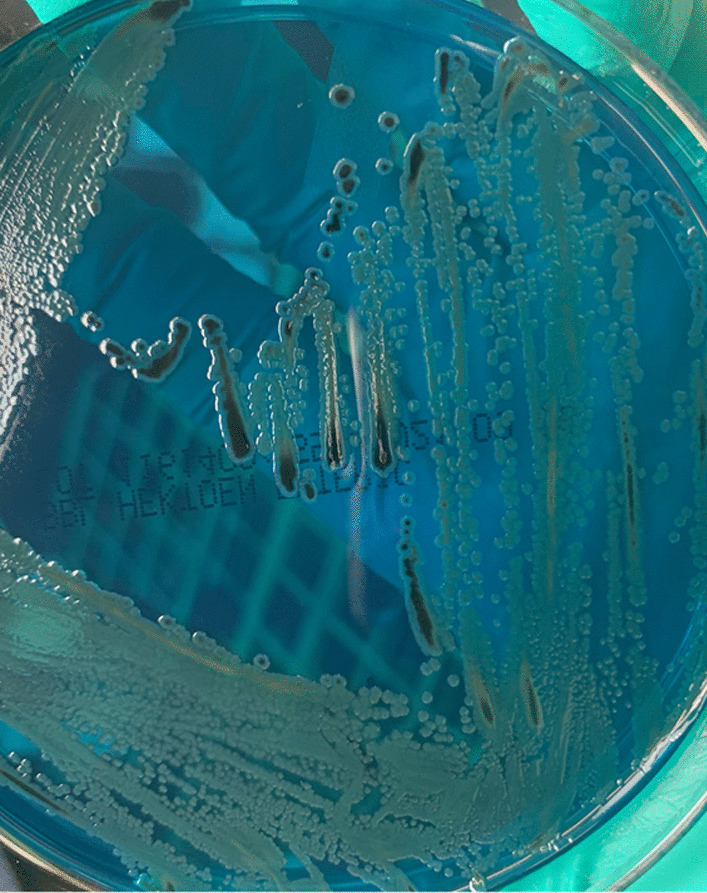
*Salmonella* spp. in selective medium (HE Agar)

*Salmonella* spp. (Fig. 8, 9 and 10) is a Gram-negative, non-spore-forming, motile, facultatively anaerobic bacillus of the family of Enterobacteriaceae. It is defined by its ability to use citrate as a carbon source, lysine as a nitrogen source, and produce H_2_S on triple agar [6]. It is not a free-living organism in the environment and has historically been a pathogen of meat or poultry products but has recently been associated with fresh and manufactured produce [7]. *Salmonella enterica* has a distinct serotype known as Choleraesuis, a highly pathogenic organism to humans associated with disseminated, systemic infections with little involvement of the intestinal tract [2].

Recent investigations on this particular serotype have revealed several suspected mechanisms for their pathogenicity. The Choleraesuis virulence plasmid (pSCV) harbors multiple factors and genes demonstrating resistance to various antibiotics such as sulfonamides and ampicillin. [8]. *S. enterica* serotype Choleraesuis infections account for a small percentage of published studies in the USA compared with reported cases from serotype Enteritidis or Typhi [9]. Extraintestinal focal infections in adults with bacteremia have been found in patients with several risk factors, such as hematological malignancy, liver cirrhosis, systemic lupus erythematosus, chronic renal impairment, and peptic ulcer disease. Approximately 20% of the patients had disseminated infections such as septic arthritis, cutaneous infection, spontaneous bacterial peritonitis, and pneumonia [10].

Joint and bone infections due to nontyphoidal *Salmonella* do occur and have been reported in the literature [17]. However, primary chest wall abscesses and costochondritis from these organisms are rare [11]. This type of infection can occur from disseminated infection or related to an open trauma or surgery. A case of chest wall abscess due to *Salmonella enterica* serovar Dublin, another nontyphoidal serovar, has been reported in a patient with a history of uncontrolled acquired immunodeficiency syndrome (AIDS) (CD4 count of 71 cells/uL) [12]. The patient underwent surgical drainage and multiple courses of antimicrobial therapy with cefotaxime (2.25 g IV every 12 hours), moxifloxacin (0.4 g IV daily), and later with biapenem (0.3 g IV every 8 hours). Treatment was given for 3 weeks with repeat CT of the chest showing substantial improvement of the abscess. Another case involved a young male with no past medical history who presented with a neck abscess due to *S. enterica* serotype Choleraesuis that required surgical drainage [13]. After leaving against medical advice, he later presented back for repeat drainage and was initially treated with levofloxacin (500 mg daily), which was later switched to ceftriaxone (2 g every 12 hours) based on antibiotic susceptibility test results. Other reported cases of extraintestinal infections were pleural empyema, fatal pneumonia, and iliopsoas abscess in immunocompromised hosts without documented bacteremia [14–16].

Treatment for nontyphoidal *Salmonella* infections depends on the type and severity of infection. In general, acute gastroenteritis is often self-limited and rarely requires antibiotic treatment. Salmonella bacteremia usually requires 10 to 14 days of treatment. More severe conditions such as meningitis may require up to 4 weeks of treatment, and more prolonged therapy is generally given for endovascular (4–6 weeks) or bone infections (6–8 weeks). Immunocompromised patients may also require more prolonged therapy [1]. In severe infections requiring antibiotic therapy, antibiotic susceptibility testing is often necessary for effective treatment due to increasing reports of drug resistance to ampicillin, chloramphenicol, fluoroquinolone, and trimethoprim–sulfamethoxazole. [18].

Our case demonstrates a case of a nontyphoidal *Salmonella* chest wall abscess and costochondritis without an obvious source. We suspect the most likely scenario was a dissemination to the traumatized chest wall from an asymptomatic gastrointestinal infection with transient bacteremia [1]. This possibly would explain her subjective fevers, chills, and night sweats 3 months before her chest wall abscess diagnosis with negative blood cultures. The mechanical fall did not result in any open wounds, and her diabetes mellitus was well controlled; she is not known to be immunocompromised, although she received a short course of steroids for acute COVID-19. It is unclear if this affected her clinical course or put her at higher risk of disseminated infection.

## Strengths and limitations

The strengths of the case report include appropriately obtaining aspirate and drainage fluid cultures to identify and confirm the organism and allowing targeted antibiotic therapy. Other appropriate work-ups include blood cultures to evaluate for bacteremia, targeted antimicrobial therapy based on antibiotic susceptibilities, close clinical follow-up, and continued coordination of care with thoracic surgery to enable source control of the infection. Limitations of the case include the patient’s limited recall of events related to factors that can possibly explain the source of *Salmonella* infection. Although it would likely be low yield as the patient had no gastrointestinal symptoms, we also note that obtaining stool studies, including cultures, might have helped to support our investigation of the original infection from a gastrointestinal source.

## Conclusion

We report a rare case of chest wall abscess with costochondritis due to *S. enterica* serotype Choleraesuis in an otherwise immunocompetent individual without a documented history of bacteremia. The condition persisted despite a series of targeted antibiotic therapy and resolved only after surgical intervention. Extraintestinal infections due to nontyphoidal *Salmonella*, including a chest wall abscess with costochondritis, can occur particularly in traumatized areas. Diagnosis is often accomplished by clinical evaluation and culture of the affected area. Treatment usually involves appropriate antibiotic therapy guided by antibiotic susceptibility testing but may require surgical intervention to achieve source control and cure.

## Data Availability

Not applicable.

## References

[1] Chiu CH, Su LH, Chu C (2004). *Salmonella enterica* serotype *Choleraesuis*: epidemiology, pathogenesis, clinical disease, and treatment. Clin Microbiol Rev.

[2] Blaser MJ, Feldman RA (1981). From the centers for disease control. *Salmonella bacteremia*: reports to the Centers for Disease Control, 1968–1979. J Infect Dis.

[3] Dhanoa A, Fatt QK (2009). Non-typhoidal *Salmonella *bacteraemia: epidemiology, clinical characteristics and its' association with severe immunosuppression. Ann Clin Microbiol Antimicrob.

[4] Harvey AM (1937). *Salmonella* suipestifer infection in human beings: review of the literature and report of twenty-one new cases. Arch Intern Med (Chic).

[5] Wu PC, Khin MM, Pang SW (1985). *Salmonella osteomyelitis*. An important differential diagnosis of granulomatous osteomyelitis. Am J Surg Pathol..

[6] Mandell, Douglas, and Bennett's principles and practice of infectious diseases. Philadelphia:Elsevier/Saunders, 2015.

[7] Jorgensen JH, Pfaller MA, Carroll KC Manual of clinical microbiology. ASM Press. 2015

[8] Chu C, Chiu CH, Wu WY, Chu CH, Liu TP, Ou JT (2001). Large drug resistance virulence plasmids of clinical isolates of *Salmonella enterica*
*serovar*
*choleraesuis*. Antimicrob Agents Chemother.

[9] Chen PL, Wu CJ, Chang CM, Lee HC, Lee NY, Shih HI, Lee CC, Ko NY, Wang LR, Ko WC (2007). Extraintestinal focal infections in adults with *Salmonella enterica* serotype choleraesuis bacteremia. J Microbiol Immunol Infect.

[10] Chen YH, Chen TP, Lu PL, Su YC, Hwang KP, Tsai JJ, Cheng HH, Peng CF (1999). *Salmonella choleraesuis* bacteremia in southern Taiwan. Kaohsiung J Med Sci.

[11] Sakran W, Bisharat N (2011). Primary chest wall abscess caused by *Escherichia coli* costochondritis. Am J Med Sci.

[12] Wang W, Chen Y, Chi Y, Huang J, Chen W, Hu Z (2020). Chronic chest wall abscess caused by *Salmonella enterica* subsp. enterica serovar Dublin infection in an HIV-infected patient: a case report and literature review. Radiol Infect Dis.

[13] Sugimoto R, Suzuki H, Nei T, Tashiro A, Washio Y, Sonobe K, Nakamura Y, Wakayama N, Inai S, Izumiya H (2017). Neck abscess due to *Salmonella choleraesuis*: case study and literature review. JMM Case Rep.

[14] Jiang LB, Zhu YH, Yao YF, Xu J, Wang Z (2012). Pyopneumothorax caused by *Salmonella choleraesuis*: a case report and review of the literature. Zhonghua Jie He He Hu Xi Za Zhi.

[15] Samonis G, Maraki S, Kouroussis C, Mavroudis D, Georgoulias V (2003). *Salmonella enterica* pneumonia in a patient with lung cancer. J Clin Microbiol.

[16] Aoyama M, Nemoto D, Matsumura T, Hitomi S (2015). A fatal case of iliopsoas abscess caused by *Salmonella enterica*
*serovar*
*Choleraesuis* that heterogeneously formed mucoid colonies. J Infect Chemother.

[17] Sy AM, Sandhu J, Lenox T (2013). *Salmonella enterica* serotype choleraesuis infection of the knee and femur in a nonbacteremic diabetic patient. Case Rep Infect Dis.

[18] Humphrey T (2001). *Salmonella typhimurium* definitive type 104 A multi-resistant *Salmonella*. Int J Food Microbiol..

